# Determinants of long-acting family planning utilization among reproductive-age women in Ethiopia: further analysis of recent demographic and health survey data

**DOI:** 10.3389/fgwh.2025.1480509

**Published:** 2025-04-10

**Authors:** Melsew Setegn Alie, Gossa Fetene Abebe, Yilkal Negesse, Desalegn Girma

**Affiliations:** ^1^ Department of Public Health, School of Public Health, College of Medicine and Health Science, Mizan-Tepi University, Mizan-Aman, Ethiopia; ^2^ Department of Midwifery, College of Medicine and Health Science, Mizan-Tepi University, Mizan-Aman, Ethiopia; ^3^ Department of Public Health, College of Medicine and Health Science, Debre-Markos University, Gojjam, Ethiopia

**Keywords:** family planning, long-acting family planning, FP, GEE, modeling, Ethiopia

## Abstract

**Background:**

Despite advancements in modern contraceptive use in Ethiopia, the uptake of long-acting family planning services remains low due to various factors. To our knowledge, there is currently no national evidence regarding the prevalence of long-acting family planning methods. Therefore, this study aimed to identify the determinants of long-acting family planning utilization among women of reproductive age in Ethiopia.

**Method:**

A secondary data analysis was conducted using the 2019 Ethiopian Demographic and Health Survey data. The data were extracted from the child record file using STATA version 15. A total of 4,782 reproductive-age women were selected for the study. After applying appropriate weighting, generalized estimating equation modeling was performed using the xtgee command in STATA. Model selection was based on the quasi-likelihood criteria, and model fitting was carried out using two proposed working correlation structures: exchangeable and independent. The generalized estimating equations modeling of the study parameters was assessed accordingly.

**Result:**

The magnitude of long-acting family planning utilization in this study was 8.6 [95% confidence interval (CI): 7.8–9.4]. Determinants of long-acting family planning utilization were age of women 40–49 years [odds ratio (OR) = 1.87, 95% CI: 1.7–4.7], rural residence (OR = 0.47, 95% CI: 0.19–0.89], female head of household (OR = 1.67, 95% CI: 1.5–2.2), family size ≥13 (OR = 0.04, 95 CI: 0.003–0.68), and number of children aged under 5 years ≥4 (OR = 0.26, 95% CI: 0.09–0.68).

**Conclusion:**

The utilization of long-acting family planning methods among women in Ethiopia is relatively low compared to the Ethiopian government's plan. Key factors influencing this utilization include age, place of residence, head of household, family size, and number of children aged under 5 years. These findings suggest that the country should enhance the use of long-acting family planning by focusing on improving access for young and adolescent women, empowering women, and addressing the needs of households with larger family sizes. Reproductive health interventions, including family planning services, should specifically target rural, male-headed households with young women who have more than four children aged under 5 years to increase the uptake of long-acting family planning methods.

## Introduction

Unintended pregnancy is a significant global issue, contributing to 27% of maternal deaths (1). Access to comprehensive sexual and reproductive health services, including a full range of contraceptive methods, is a fundamental human right essential for overall wellbeing (2). Universal access to contraception can prevent the adverse health and socioeconomic consequences associated with unintended pregnancies. Global initiatives advocate for universal access to family planning (FP) services as a human right. Maternal health services encompass family planning, which is crucial for improving the health of mothers, infants, and children (2–4).

To reduce unintended pregnancies, long-acting family planning (LAFP) methods are a superior option (5–8). They are cost-effective and empower women to control their reproductive choices. The use of long-acting and permanent contraceptive methods can significantly increase the contraceptive prevalence rate in countries with low coverage (8). Research shows that women using short-acting contraceptives are 21 times more likely to experience unintended pregnancies compared to those using long-acting reversible or permanent methods (9). A projection for countries in sub-Saharan Africa indicated that over 1.8 million unintended pregnancies could have been averted within a 5-year period if 20% of women using oral contraceptives and injectables had switched to implants (10). In 2015, long-acting and permanent contraceptive methods, such as intrauterine devices (IUDs), implants, and sterilization, represented 56% of global contraceptive use. Specifically, 19% of married or in-union women opted for female sterilization, while 14% utilized IUDs. However, the majority of contraceptive users in Africa still rely on short-term methods (8).

In previous studies, the following determinants were identified: age (11–15); marital status (13, 16); religion (17); educational status (3, 11, 12, 14, 16–22); previous utilization of long-acting family planning methods (3, 4, 11, 19, 23); work status (12, 14, 22); information on long-acting family planning methods (3); discussions with health providers on long-acting contraceptives (4, 20, 24); spousal discussions (18, 23); family size (18); receiving counseling services on long-acting contraceptives during delivery and immediate postpartum periods (19); fertility-related characteristics, wealth index, and exposure to mass media (12); desired number of children (25); availability of family planning services (13); being told what to do if they experience side effects and knowledge of the source of long-acting family planning methods (20); method availability, future intention to become pregnant, and walking distance of more than 30 min from associated health facilities (26); high economic status and history of abortion (24); women in difficult financial situations, smokers, and women in the care of a gynecologist (15); age at first delivery and no desire for more children (17); no birth intention (23); exposure to family planning messages and no desire for more children (22); and residing in urban areas and wanting children after 2 years (16). The determinants of long-acting family planning can vary significantly by region, and recent studies at the country level, particularly the Ethiopian Demographic and Health Survey (EDHS 2019), have not thoroughly examined these factors. Up-to-date and summarized evidence on the determinants of long-acting contraceptives is crucial for understanding the challenges and establishing effective intervention mechanisms. Furthermore, insights into these determinants can inform the design of appropriate programs and strategies. Identifying these determinants allows for the development of targeted programs that address specific barriers, ultimately reducing ineffective interventions within the country. Therefore, the objective of this study was to identify the determinants of long-acting family planning utilization among reproductive-age women in Ethiopia using Demographic and Health Survey (DHS) data. Specifically, it sought to determine the prevalence and key factors influencing the use of long-acting family planning utilization in Ethiopia.

## Material and Methods

### Study design and setting

The data utilized in this study comes from the Ethiopian Demographic and Health Survey conducted in 2019. This research was carried out at the national level in Ethiopia, located in Eastern Africa. The cross-sectional survey is conducted every 5 years and covers the entire country. Ethiopia consists of nine regional states and two city administrations.

### Data source and participants

The dataset for this study was obtained from the DHS database, accessible through DHS Program after receiving approval. The DHS is conducted every 5 years to provide health indicators at both the national and regional levels, utilizing a representative sample. Its data collection tools are pretested and tailored to each participating country’s context. The EDHS-2019, the fifth iteration, was the most recent available. Relevant data were extracted from the Kid Record (KR) file to align with the study's objectives. The EDHS 2019 employed a two-stage sampling design with strata for urban and rural areas. A new dataset comprising the necessary variables was created, and the extracted data were checked for completeness, transformed, recorded, and merged using STATA version 15 for analysis. The source population included all women of reproductive age, while the study population consisted of sexually active women in a marital union or co-habiting. After excluding infecund and sexually inactive individuals, the final sample comprised 4,782 reproductive-age women. Detailed information on data collection processes and tools is available in the demographic dataset.

### Variables

#### Dependent variable

The Demographic and Health Survey defines modern contraceptive use among married women to include various methods: male and female sterilization, intramuscular injections, IUDs, contraceptive pills, implants, male condoms, lactational amenorrhea, and emergency contraception (27).

In this study, the primary outcome variable was the use of long-acting contraceptives. Contraceptive methods were categorized into two groups: long-acting methods (specifically IUDs and implants), coded as “1”; and all other methods (including short-acting and traditional methods), coded as “0.” Women using IUDs or implants were assigned a code of “1,” while those using any other method were assigned a code of “0.”

#### Independent variables

Independent factors influencing contraceptive use were selected based on prior research and the available variables in the EDHS 2019 dataset. This study included the following variables: women's age, place of residence, household wealth index, total number of living children, birth interval, number of children aged under 5 years in the household, age at first delivery, place of delivery, survival status of the last child, mode of delivery [cesarean section (C/S)], and family size.

### Data analysis

The extracted data were recoded and cleaned using STATA version 15 for further analysis. Generalized estimating equation (GEE) modeling was performed using the xtgee command. Before analysis, the data were weighted to ensure a representative sample. A descriptive analysis assessed participants’ background, obstetric, and reproductive health characteristics. GEE, based on quasi-likelihood estimation, was selected using quasi-likelihood criteria (QLC). The xtgee command was configured with a binomial family and a logit link function, utilizing exchangeable and independent working correlation structures. Variables with large *p*-values were removed sequentially, and the model was refitted until the best fit was achieved. Odds ratios with 95% confidence intervals (CIs) were used to identify statistically significant variables.

### Generalized estimated equations

GEE is used for clustered and repeated measures data. It is a non-likelihood approach that accounts for within-cluster associations using marginal correlation. The GEE model is based on a generalized linear model and the correlation structure is given as follows:

$$g(\pi_{j})=\mathrm{logit}(\pi_{j})=x_{\;j\text{ß}}^{\prime }$$

where *g*(*π_j_*) is the logit link function, *x'_j_* _=_ *n_j_*_×*p*_ is the dimensional vector of known covariate, *ß* command is configured with dimensional vector of unknown parameter, and *π_j_* is the expected value of response variable in a specific cluster, which is binomial distribution.

### Parameter estimation for GEE

The ß value in GEE is estimated by solving an equation that incorporates a working correlation matrix and a marginal variance matrix with non-zero values on the main diagonal and zeros elsewhere. The correlation structure is given as follows:

$$S(\text{ß})=\overset{M}{\underset{i=1}{\sum }}\;\left(\frac{\partial \pi j}{\partial {\text{ß}}^{\prime }}\right)[Aj1\;\;/\;2\;RjAj1\;\;/\;2\;{]}^{-1}\;\;(Yj-\pi j)=0$$

### Model building

The quasi information criterion (QIC) was used to identify significant predictor variables for long-acting family planning, as GEE is based on quasi-likelihood ratios. The optimal model was selected by choosing the one with the lowest QIC. Two types of working correlation structures—exchangeable and independent—were considered, with the exchangeable correlation proving to be the best fit.

## Results

### Background characteristics of study participants

Table 1 shows that nearly half (41.3%) of the study participants resided in the Oromia region. A significant majority (76.7%) of the participants lived in rural areas. In terms of religion, over one-third (38.8%) of the women identified as Muslim. Half (50.4%) of the women were aged 20–29 years and almost half (46.5%) of the women fell into the poor wealth category. In addition, a substantial majority (89.4%) of households were male-headed, and more than half (58.6%) had five to eight members (see Table 1).

**Table 1:** Background characteristics of the study participants in Ethiopia, 2019.

Variables	Frequency	Percent
Residence		
Urban	1,114	23.3
Rural	3,668	76.7
Maternal age		
15–19	197	4.1
20–29	2,410	50.4
30–39	1,761	36.8
40–49	414	8.7
Wealth status		
Poor	2,225	46.5
Middle	920	19.3
Rich	1,637	34.2
Sex of household head		
Male	4,273	89.4
Female	509	10.6
Family size		
≤4	1,344	28.1
5–8	2,804	58.6
9–12	590	12.3
≥13	44	1.0

### Obstetric and reproductive health characteristics

The majority of women (65.2%) give birth for the first time before the age of 18 years. Nearly half (47.5%) had three or fewer children, and a significant majority (94.7%) had one to three children at the time of data collection. In addition, 83.5% of the women had one to two children aged under 5 years. More than half (52.6%) delivered their first child at home, and 51% reported having a birth in the last 3 years (Table 2).

**Table 2:** Obstetric and reproductive health characteristics of study participants in Ethiopia, 2019.

Variables	Frequency	Percent
Age at first birth (years)		
≤18	3,119	65.2
19–24	1,404	29.4
≥25	259	5.4
Parity		
≤3	2,273	47.5
4–5	1,128	23.6
≥6	1,381	28.9
Total number of children		
None	200	4.2
1–3 children	4,527	94.7
≥4 children	56	1.1
Number of under-5 children		
None	200	4.2
1–2	3,991	83.5
≥3	591	12.4
Index child alive		
Yes	4,370	91.4
No	412	8.6
Sex of index child		
Male	2,553	53.4
Female	2,229	46.6
ANC follow up for index pregnancy		
No	831	24.2
1–3	1,054	30.8
4–6	1,389	40.5
≥7	153	4.5
Place delivery		
Home	2,487	52.6
Health facility	2,192	46.3
Others	54	1.1
Cesarean delivery		
Yes	255	5.3
No	4,527	94.7
Birth history in the last 3 years		
Yes	2,463	51.5
No	2,319	48.5
Long-acting family planning use		
Yes	410	8.6
No	4,372	91.4

### Descriptive summary of long-acting family planning use

The final model variables presented in Table 3 show that nearly half (49.3%) of women aged 20–29 years use long-acting family planning methods. In addition, women in urban areas are more likely to use long-acting family planning than those in rural areas, with usage rates of 32.0% and 22.5%, respectively. Furthermore, over half (58.2%) of women in households with five to eight members use long-acting family planning methods.

**Table 3:** Descriptive summary of long-acting family planning use among reproductive-age women in Ethiopia, 2019.

Variables	Level	Long-acting FP use	
		Yes *N* (%)	No *N* (%)
Age	15–19	14 (3.4)	183 (4.2)
	20–29	202 (49.3)	2,208 (50.5)
	30–39	158 (38.5)	1,603 (36.7)
	40–49	36 (8.8)	378 (8.6)
Residence	Rural	278 (68.0)	3,390 (77.5)
	Urban	131 (32.0)	983 (22.5)
Sex of HH head	Female	31 (7.6)	478 (10.9)
	Male	378 (92.4)	3,895 (89.1)
Family size	≤4	133 (31.6)	1,211 (27.7)
	5–8	245 (58.2)	2,559 (58.6)
	9–12	30 (7.1)	559 (12.8)
	≥13	13 (3.1)	40 (0.9)
Number of under-5 children	None	21 (5.7)	178 (4.1)
	1–3	330 (89.9)	4,130 (94.7)
	≥4	16 (4.4)	55 (1.2)

### Analysis of GEE

The GEE model-building strategy begins by fitting a model that includes all possible factors in the data. This process considers two different working correlation assumptions: exchangeable and independent. The full model for the probability of getting long-acting family planning use of *i*th women from *j*th cluster (л_ij_) was fitted as follows:

Logit(л_ij_) = ß0 + ß1Tigra + ß2Afar + ß3Amhara + ß4Oromo + ß5Somali + ß6Bishangul + ß7SNNPR + ß8Gambella + ß9Harari + ß10Dirediwa + ß11Urban + ß12protestant + ß13Muslim + ß14others + ß15 20–29 years + ß16 30–39 years + ß17 40–49 years + ß18Middle wealth + ß19rich wealth + ß20male HH head + ß21 5–8HH member + ß22 9–12 HH member + ß23 ≥ 13 HH member + ß24 19–24 years at first birth + ß25 ≥ 25 years at first birth + ß26 parity 4–5 + ß27 parity ≥6 + ß28total number of children 1–3 + ß29 total number of children ≥4 + ß30 number of under five children 1–2 + ß31 number of under five children ≥3 + ß32 index child alive (Yes) + ß33 sex of index child(male) + ß34ANC 1–3 + ß35 ANC 4–6 + ß36 ANC ≥7 + ß37 place of delivery(health facility) + ß38 place of delivery (other) + ß39C/S delivery(yes) + ß40 birth in last three year(yes). After reducing the variables, those with the smallest *p*-value were refitted for the best model. Variables with large *p*-values were removed first. The final model included age, residence, sex of the household head, household size, and number of children aged under 5 years. Empirical and model-based standard errors were calculated for the two proposed working correlation structures. The exchangeable correlation assumption was deemed appropriate, as the two standard errors were closely aligned with the correlation parameter. Therefore, the final generalized estimating equation model for long-acting family planning use is as follows:

Logit(л_ij_) = ß0 + ß1 20–29 years + ß2 30–39 years + ß3 40–49 years + ß4Urban + ß5 female HH head + ß6 5–8family size + ß7 9–12 family size + ß9 ≥ 13 family size + ß10 total number of under five children 1–3 + ß11 total number of under five children ≥4. For ease of interpretation, odds ratio was used instead of coefficient estimates (ß). The QIC value for the saturated model was 3,049.067, while the QIC value of the final model was 2,817.803. The exchangeable working correlation model was the best fit compared to the independent model, as determined by the smallest QIC value. The most parsimonious model included age, sex of the household head, residence, family size, and number of children aged under 5 years. The final GEE model, along with their standard error, is shown in Table 4. As shown, the determinants of long-acting family planning use were age, residence, sex of the household head, family size, and number of children aged under 5 years in the house (Table 4).

**Table 4:** Parameter odds ratio and their corresponding empirically corrected standard error (SE) with *p*-values of the final model in the Ethiopian study.

Variables	Level	Odds ratio	SE	95% CI	Pr>|Z|
Intercept		0.10	0.02	0.07–0.15	0.000
Age	15–19	Ref			
	20–29	1.33	0.13	0.61–0.2.89	0.47
	30–39	1.36	0.60	0.57–3.24	0.48
	40–49	1.87	0.08	1.7–4.7	0.02
Residence	Rural	0.47	0.09	0.19–0.89	0.009
	Urban	Ref			
Sex of HH head	Female	1.69	0.013	1.5–2.2	0.005
	Male	Ref			
Family size	≤4	Ref			
	5–8	0.95	0.22	0.6–1.5	0.82
	9–12	0.62	0.27	0.27–1.44	0.27
	≥13	0.04	0.06	0.003–0.68	0.02
Under-5 children in house	None	Ref			
	1–3	0.61	0.63	0.27–1.41	0.26
	≥4	0.26	0.13	0.09–0.68	0.006
QIC = 2,817.803					

Ref, reference; QIC, quasi-likelihood criteria; CI, confidence interval.

## Discussion

This study used data from the EDHS 2019 to identify the determinants of LAFP utilization among reproductive-age women in Ethiopia. Understanding these determinants is crucial for early intervention and for enhancing access to and the quality of family planning services.

The prevalence of LAFP utilization among reproductive-age women in this study was found to be 8.6% (95% CI: 7.8–9.4). This finding aligns with similar studies conducted in Debre Tabor, northwest Ethiopia (9.2%) (28) and Goba, southeast Ethiopia (8.7%) (29), while it is lower than the study conducted in Debre Markos, northwest Ethiopia (19.5%) (30), Mekelle, northern Ethiopia (12.3%) (31), western Ethiopia (20%) (32), Bahir Dar, Northwest Ethiopia (27.5%) (23) Mahabad, Iran (27.7%) (33), Kampala district, Uganda (31.7%) (34), and South Africa (34%) (35). This variation could be attributed to the inclusion of both urban and rural areas in the present study, with a large and diverse population with sociodemographic differences among study participants. Another possible reason is the varying levels of acceptance in the community of family planning. In Uganda and South Africa, there may also be differences in family planning counseling during service delivery. However, the use of LAFP methods in this study is higher than reported in studies conducted in Jinka, southern Ethiopia (7.3%) (36) and central Ethiopia (3%) (37). This difference could be due to sociodemographic variations, differences in in community perceptions toward LAFP, and the time between the studies. In addition, the larger sample size in the current study may be a factor. The highest utilization rates of LAFP were observed in the Amhara, Addis Ababa, and Oromia regions, which compares with previous studies (38–40). This may be attributed to the effective implementation of health extension programs and the integration of family planning services. In addition, as the capital city, Addis Ababa probably has increased access to healthcare facilities and increased availability of family planning information.

The best-fitting model identified several determinants of long-acting family planning utilization, including maternal age, residence, sex of the household head, family size, and the number of children aged under 5 years in the household. Women aged 40–49 years in Ethiopia were found to be 1.87 times more likely to utilize long-acting family planning methods compared to those aged 15–19 years. This finding aligns with studies conducted in sub-Saharan Africa (12), Oromia, Ethiopia (11), Arba Minch, Ethiopia (13), Arsi, Oromia, Ethiopia (14), and France (15), all of which indicate that increased maternal age is associated with higher rates of long-acting family planning use. This trend may be attributed to the challenges faced by adolescents and young adults in accessing family planning services and their lower motivation to seek these services.

Furthermore, women residing in rural areas are 53% less likely to utilize long-acting family planning methods compared to their urban counterparts, with an odds ratio of 0.47 (95% CI: 0.19–0.89). This finding aligns with studies conducted in Southern Ethiopia (41) and Kenya (42), which also demonstrated that women in rural regions are less likely to adopt long-acting family planning methods than in urban areas. The lower utilization of these methods among rural women can be attributed to several factors, including limited access to healthcare services, lack of knowledge about long-acting family planning methods, cultural barriers, partner influence, concerns about potential side effects, and lower levels of education, which hinder informed decision-making about contraception. In contrast, women in urban areas generally have better access to healthcare services and information, enabling them to make more informed choices regarding family planning. In addition, this disparity may be due to differences in access to family planning services and information between rural and urban women in Ethiopia.

The study highlights that Ethiopian women who lead their households are significant determinants of long-acting family planning use among reproductive-age women in Ethiopia. Specifically, the odds of utilizing long-acting family planning methods are 1.69 times higher in female-headed households compared to male-headed ones. This trend can be attributed to the increased empowerment of women, which enhances their ability to control resources within the household. In addition, family size and the number of children aged under 5 years are critical factors influencing the use of long-acting family planning. Women in Ethiopia with a family size of 13 or more are 96% less likely to use long-acting family planning methods compared to those with a family size of four or fewer. This finding aligns with research conducted in western Ethiopia (18), suggesting that cultural support for larger families may reduce the motivation to adopt family planning. Moreover, larger family sizes can limit access to family planning resources and decrease the time available for women to seek such services.

## Strengths and limitations of this study

• This is a country-level study which allows for generalization to the broader population.
• It has a large sample size, which provides greater precision in the estimates.
• The study participants were from both urban and rural areas, allowing the findings to be generalized to the source population across the country.
• Generalized estimated equation modeling was used, which offers working correlations.
• The cross-sectional nature of the study does not show cause-and-effect relationships between the predictors and outcome variables.
• The study does not account for cultural, availability, and accessibility factors that can significantly influence the utilization of family planning services. Furthermore, the study did not include qualitative data, which may have given different results. Future research should consider these factors and readers should consider these limitations when reviewing this paper.

## Conclusion

The utilization of long-acting family planning methods among Ethiopian women in this study is relatively low compared to previous studies conducted in other countries and the Ethiopian government's target of 55% (43). Factors influencing the use of long-acting family planning include women's age, place of residence, the sex of the household head, family size, and the number of children aged under 5 years in the household.

To enhance the utilization of long-acting family planning services, it is essential to improve both access and quality. Interventions should specifically target male-headed households with younger women and larger family sizes. The Federal Ministry of Health should focus on increasing the utilization of long-acting family planning methods among rural male-headed households, particularly those with a higher number of children aged under 5 years.

In addition, researchers are encouraged to qualitatively explore the reasons behind the low usage of long-acting family planning among Ethiopian women. Tailored, multifaceted interventions and the strengthening of primary healthcare services could significantly improve the adoption of long-acting family planning methods. Special emphasis should be placed on younger women from larger families and those living in male-headed households.

## Data Availability

The datasets presented in this study can be found in online repositories. The names of the repository/repositories and accession number(s) can be found in the article/Supplementary Material.
